# A Cost-Consequences Analysis of a Primary Care Librarian Question and Answering Service

**DOI:** 10.1371/journal.pone.0033837

**Published:** 2012-03-19

**Authors:** Jessie McGowan, William Hogg, Jianwei Zhong, Xue Zhao

**Affiliations:** 1 Faculty of Family Medicine, University of Ottawa, Ottawa, Ontario, Canada; 2 Faculty of Medicine, University of Ottawa, Ottawa, Ontario, Canada; 3 Institute of Population Health, University of Ottawa, Ottawa, Ontario, Canada; 4 C.T. Lamont Primary Health Care Research Centre, Élisabeth Bruyère Research Institute, Ottawa, Ontario, Canada; 5 Department of Economics, Carleton University, Ottawa, Ontario, Canada; Yale University School of Medicine, United States of America

## Abstract

**Background:**

Cost consequences analysis was completed from randomized controlled trial (RCT) data for the Just-in-time (JIT) librarian consultation service in primary care that ran from October 2005 to April 2006. The service was aimed at providing answers to clinical questions arising during the clinical encounter while the patient waits. Cost saving and cost avoidance were also analyzed. The data comes from eighty-eight primary care providers in the Ottawa area working in Family Health Networks (FHNs) and Family Health Groups (FHGs).

**Methods:**

We conducted a cost consequences analysis based on data from the JIT project [1]. We also estimated the potential economic benefit of JIT librarian consultation service to the health care system.

**Results:**

The results show that the cost per question for the JIT service was $38.20. The cost could be as low as $5.70 per question for a regular service. Nationally, if this service was implemented and if family physicians saw additional patients when the JIT service saved them time, up to 61,100 extra patients could be seen annually. A conservative estimate of the cost savings and cost avoidance per question for JIT was $11.55.

**Conclusions:**

The cost per question, if the librarian service was used at full capacity, is quite low. Financial savings to the health care system might exceed the cost of the service. Saving physician's time during their day could potentially lead to better access to family physicians by patients. Implementing a librarian consultation service can happen quickly as the time required to train professional librarians to do this service is short.

## Introduction

Access to family physicians (FPs) is a major concern for the Canadian health care system. Despite increased medical school enrollment, more medical students choosing family medicine, and programs to facilitate the licensing of foreign medical graduates, it will take years to redress the shortage. Recently, in the province of British Columbia, the northern medical program, a program funded to train students in northern practices in hope that the graduates will practice rurally, was criticized for the fact that only five out of the first call of 24 graduates in 200 started a practice in rural [2]. Canada remains near the bottom of the Organization for Economic Cooperation and Development countries for the numbers of medical students and practicing doctors per capita [3]. Improving the efficiency and effectiveness of the existing FP workforce could help address the problem. Using other health professionals to participate in patient care is a promising strategy to increase system capacity. Librarians could be part of the inter-professional effort. A project called the “Just-in-time librarian consultation service (JIT)” was designed to test if the provision of a question and answering service could improve the efficiency and effectiveness of FPs by saving them time.

FPs have many questions that arise while seeing patients. Clinical questions arise on a daily basis for physicians as they are faced with an amazing variety of illness every day. When a FP sees a new patient, the conditions of the patient are usually undifferentiated and disorganized. It is not possible that FPs will know everything about the many different subjects covered in a typical day at work. In practice physicians use many methods to provide effective patient care. For example, they may deal with uncertainty by having the patient return for a second appointment to learn how the condition is evolving. They may send the patient to a specialist for consultation, consider ordering tests or try a medication. They could consult their own library and reference files for the information needed, while the patient waits. If they do not have time during the visit, they could ask the patient to make a follow up appointment. They could also have a corridor consultation with a colleague or send a request to a librarian for a literature search on a particular topic. There are many obstacles to answering questions and Ely found fifty-nine obstacles in a qualitative study [4]. The categories included recognizing an information need, formulating questions, information seeking, answer formation, and applying answers to patient care. A review of the literature by Davis identified that the frequency with which doctors asked questions derived from patient care ranged from 0.16 to 1.27 questions per patient [5]. However, while physicians have questions when they are seeing patients, they only pursue answers to about 30% and were significantly more likely to pursue answers to their clinical questions when they believed that their definitive answers to those questions existed [6].

The JIT service has been described in detail elsewhere [1], [7], [8]. Briefly, in the JIT study, participants were provided with a handheld device (BlackBerry™) at no cost so they could send clinical questions to the library service between 9 a.m. and 5 p.m., Monday to Friday. When a question was allocated to the intervention group, their question was answered by the service. When the question was allocated to control group, no answer was provided (though a message stating this was sent immediately) and therefore the participants had to find the answers themselves. The investigators and librarians were blinded to allocation of all questions (and librarians answered all questions, regardless of allocation).

The results of the randomized controlled trial showed that providers who used the service saved time when questions were answered by a librarian and there was a reduction in the number of follow-up visits [1]. Other investigators have also shown the use of a question and answer service to provide information to be effective [9], [10], [11], [12], [13], [14], [15]. In this paper, we evaluate the JIT service using a cost consequences analysis (CCA), an approach that is readily understood and applied by healthcare decision-makers [16], [17].

## Methods

### Ethics statement

The original randomized controlled trial (RCT) [1] received with ethical approval from the Ottawa Hospital Research Ethics Committee, Ottawa, Ontario, Canada. All participating participants signed consent forms as part of the enrolment process for the study.

### Study setting and participants

JIT targeted primary care providers in the area of Ottawa, Ontario, Canada. The providers worked in Family Health Networks (FHNs) and Family Health Groups (FHGs), two new models of primary care service delivery in Ontario and ran from October 2005 to April 2006. Data from the run-in period, which preceded the trial, was not included. JIT included 88 primary care providers (93% FPs, 6% nurses, and 1% residents) but for this cost consequence analysis only the data from physicians was used, as the percentage of responses from nurses and residents was too low for their results to be interpreted meaningfully. Data was used from the intervention group where the librarian answered questions and the answers returned to the physician.

### Analytic Overview

The CCA was conducted from the Ontario government's perspective. All costs were expressed in Canadian dollars using actual costs from the data collection period. (see Table 1).Two types of direct costs were included: fixed and variable. The fixed costs were defined as expenses that do not change in proportion to the amount of medical services provided (i.e. equipment). The variable costs included those, which vary with the quantity of medical services provided and the duration of the intervention (i.e. administrative costs). Indirect costs, intangible costs, and costs related exclusively to the conduct of the research were excluded. Fixed costs, such as equipment that could be used post-project, were amortized [19].

**Table 1:** Resources utilization for JIT Service (2006 Canadian dollars).

	CAD ($)	Percentage	Reference
Total direct cost for JIT per month	7,728.6	100%	
*Training* 1	46.3	0.6%	[19]
Labor (Librarian, 2.26 FTEs, includes 10% benefits)	5,769.4	74.7%	[7]
*Equipment* 2	1,407.1	18.2%	[19]
Project software and technical support	1,009.9		
Handheld and wireless access	335.1		
Office supplies and information resources	20.4		
Other	41.8		
*Administration*	505.8	6.5%	[7]
Traveling	118.5		
Administrative Overheads	387.3		
Total questions sent to the JIT in this study	1,417		[7]
Average Costs Per Question in this study^3^	38.2		[7]
Average Costs Per Question at Capacity^4^	5.7		[18]

1 Training costs had been amortized over a three-year horizon, using 5% as the discount rate;

2 Costs of equipment had been amortized over a five-year horizon, using 5% as the discount rate;

3 In this study, on average 202 questions were sent monthly to the JIT; and

4 Capacity has been estimated to be 1,357 questions, given a 90% target utilization rate of the service.

The fixed costs were classified using the following categories: 1) training; 2) librarian labor; 3) equipment; and 4) administration. The number of librarians employed for the study was 2.26 and 80% of their actual salaries (including 10% of benefits) was attributed to the cost of service provision while the remainder was attributed to research and evaluation of the service. The JIT librarians had the time to answer more questions. The number of librarians required to deliver this service was estimated by determining the maximum number of questions answerable by a librarian. The method proposed by Ingolfsson and Gallop was used to estimate the number of questions that would reach the target utilization rate for a program [18].

Five categories were used to cost equipment: 1) project software and technical support; 2) handheld devices and wireless access; 3) office supplies (i.e., printing, paper, etc.); 4) information resources for the librarians; and 5) other. These were amortized over a five-year period [19]. Only 80% of the costs of project software and technical support were included as service costs, with 20% of the resources estimated as research costs.

Each participant was provided with a handheld device. For the equipment costs, we included the handheld purchase costs and the wireless access fees. Administration costs included overhead such as traveling costs. Fifty percent of traveling costs were attributed to the program delivery, while the other half was related to research activities and program evaluation.

### Consequences for Family Physicians using a librarian consultation service

To test whether or not this service could allow FPs to see more patients, the minutes saved per question asked and the number of questions asked per month was measured. The average number of clinical hours worked by FPs and the average number of patients seen per FP were also included.

FPs could use their time saved from participating in the JIT project in a variety of different ways. For instance, a FP could use the time to see more patients, to spend more time with patients, to take a break, or to go home early. Given this variation, an upper and lower bound on the number of extra patients that could be seen and include a scenario based analysis was proposed.

It was recognized that many of the study parameters could vary in an ongoing program. For example, physicians working in computerized settings might choose to send their questions to the librarians from their desktop or workstation. Accordingly, we computed the costs with and without the handheld devices and used different discount rates (3%, 5%, and 10%) to amortize the equipment and training costs.

### Cost saving of using a librarian consultation service

The impact of timely answers for the clinical questions to estimate the monetary value of the JIT service to the health care system was examined (see Table 2). A physician's time is a limited health care resource. Benefit is generated when a physician's time is spent more effectively. By using the JIT service, a physician receiving the answer to a clinical question would save the amount of the time he or she would spend finding the answer him or herself. In the case when he/she is asking another physician for help, the time of the other physician is also saved. For each question, we calculated that, the cost saving is equal to the time saved multiplied by the average hourly wage rate of the physician. Replacing the more expensive time of the physician with the less expensive librarian's time has the potential to save the system money.

**Table 2:** Cost saving and cost avoidance from JIT Service (2006 Canadian dollars).

	CAD ($)	Range ($)	Reference
**Average cost saving and cost avoidance per question**	11.55	10.63–12.47	
**Cost saving per question**	10.58		
Physician time saved from less frequently look up answer by themselves	10.45	9.64–11.25	[7], [20], [21]
Physician time saved from being less consulted by clinic members and other physicians	0.13	0.12–0.14	[7], [20], [21]
**Cost avoidance per question**	0.97		
Less referral to specialist	0.97	0.87–1.08	[21]
Fewer diagnostic tests ordered	Not significantly different between control and intervention groups		
Physician time saved from less follow up appointment	Not significantly different between control and intervention groups		

Secondly, by getting timely answers for the clinical questions, the JIT project was able to show that a physician can reach a diagnosis and recommend therapy with fewer diagnostic tests. The cost avoidance of these laboratory and imaging tests is calculated by multiplying the reduction in tests order after having used the JIT service by the average cost of tests. The average cost of the three most frequently ordered laboratory (CBC, Electrolytes and Glucose test) and imaging tests (chest X-ray, mammogram and ECG) in Ontario was used to approximate the average cost for tests [20]. Similarly the JIT project was able to show that a physician using the service required patients to return for follow up appointments less often and patients were referred to specialist less frequently.

### Cost avoidance of using a librarian consultation service

Cost avoidance from reduced patient follow-up visits for that clinical problem is calculated by multiplying the reduced visit rate by the cost of a visit. The cost avoidance from less frequent referrals is calculated by multiplying the reduced referral rate by the average cost of a specialist visit. The average fee for visits to the top four specialist services (to General Surgery, Obstetrics/Gynecology, Orthopedic Surgery, and Dermatology) in Ontario was used to approximate the average cost of a specialist visit [20].

## Results

### Capacity of the librarian consultation service

A total of 1,417 questions were asked by the family physicians during a seven-month period. For each FP, this is approximately two questions per month, or 24 questions per year. Because this was a research trial, there was idle service capacity and the librarians could have answered more questions. Based on a 7.5-hour day, the librarians were answering clinical questions about 13% of the time. In the context of a functioning program, if we assume the librarians were to answer questions 90% of their time, and then a service of this size could handle 1,357 questions per month, following a Poisson distribution [18].

### Cost related to running a librarian consultation service

The direct costs per month of for providing the librarian consultation services are shown in Table 1.

### Human resource costs

Human resources accounted for 75.3% of the total direct costs.

### Equipment and administration costs

Equipment and administration costs were 18.2% and 6.5%, respectively. The direct cost per question was $38.20. This cost is an overestimate since the librarians and other resources were not fully used. At 90% capacity, direct costs per questions would be sharply reduced to $5.70.

### Consequence of saving physicians' time

By using a librarian consultation service, physicians could save time and the consequences of this saved time can allow them to see additional patients. Below, we present three scenarios for how saved time could be used.

### Time savings (seeing additional patients 1)

The average time for physicians to find the answer to their questions themselves (when the question was assigned to the control group) was 20.29 minutes. According to the 2007 National Physician Survey, the average FP booking time interval is 13.6 minutes [21]. If FPs saw one additional patient whenever the JIT service saved them time, then each FP could each see 24 extra patients per year.

### Ability to see additional patients based on time savings (seeing additional patient 2)

Another way to calculate the capacity to see extra patients is to take the average time for participants to respond to control questions (20.29 minutes) and relate this to the amount of time physicians spend seeing patients. According to the National Physicians survey, a FP spends on average 7830 minutes per month on direct patient care. Saving 40.5 minutes per month by asking two questions to the JIT service represents a 0.52 percent time saving. This is a small increase but it is significant if it is applied nationally.

### Ability to see additional patients based on time savings (increased physician capacity)

In 2006, there were 31,989 active FPs in Canada [21]. If we assume that 42% use this service (this being the response rate of this study) then about 12,796 FPs would potentially benefit from this new service. A 0.52 percent timesaving is equivalent to 135 family physicians working full time. This is equivalent to the entire graduating class of a Canadian medical school being added to the primary care sector of the health care system, with the capacity to look after the population of a city the size of Saskatoon or Halifax. This increase in capacity could happen very quickly as the JIT service is scalable and librarians are available.

### Librarian and direct costs to deliver a national service

A primary cost for this service was the costs of employing librarians. If we consider a 90% service utilization rate, with two questions per month per FP, then 70 librarians would be required. The librarians and other direct costs would cost $216,255 per month (or $2,595,071 per year). Overall, a JIT program could make a modest contribution to the effort of reduce waiting time for family physician services in our health care system. Choosing different discount rates (3% and 10%) for the amortization did not appreciably change our results. The lack of impact is attributed to the high percentage of costs being labor costs.

### Cost saving of using the librarian consultation service

As shown in Table 2, the expected cost saving each time the physician used the JIT service was determined as $10.58. First, the hourly wage of the FP was approximated based on the average FP booking time interval per visit (13.6 minutes from 2007 National Physician Survey) and average fee for per visit. The 2006 benefit schedule for physician services in Ontario suggests $30 as the fee for an average visit for a FP. Considering the average length of patient encounter is 13.6 minutes for FPs, we calculate the average hourly wage rate for FP in our study as about $132. Note that this is the gross wage rate of the FP, given that they still need to pay overhead costs of their practices. But it is the “gross” that matters for the government's health care expenditure [22]. Secondly, we use the average time for physicians to respond to control questions (20.29 minutes) to measure the time saved. A 95% confidence interval (18.72 to 21.86) minutes is used to perform the sensitivity analysis. The probability of having this time saving is 23.4%, as the physician did not always look up the answer when the question was assigned to the control group (40.5%), and sometimes looked up the question even when the librarian provided an answer (17.1%).

The cost saving also came from physicians being less frequently asked by their colleague for the clinical questions. Once asked, the chance that they could give a quicker response is high since their colleagues are more likely to have pre-selection before consult for help. We assume they spend only one fourth of the time that should be used to respond to the control question (20.29/4 minutes). The probability of getting this time saving is 1.2% (3.8%-2.6%). Given the small size of the probability, the cost saving from this aspect is trivial (about 13 cents).

### Cost avoidance of using the librarian consultation service

The cost avoidance resulting from fewer referrals to specialists was estimated in Table 2 as 97cents. In the estimation, we use the average fee for specialist visits for four frequently consulted specialties at $80.69. According to the benefit schedule of the health insurance in Ontario, the fees for first consultation with the specialists are as follow: General Surgery ($90.3), Obstetrics/Gynecology ($77.2), Orthopedic Surgery ($83.1), and Dermatology ($72.15). The average fee is then $80.69 [22]. The probability of having this cost avoidance is 1.2% (3%-1.8%), obtained by comparing the percentage of chance to arrange referrals by the physician when the clinical questions are assigned to the control group (3%) or the intervention group (1.8%).

### Cost savings and cost avoidance per question

Overall, the average cost savings and cost avoidance for each question with the JIT service is estimated to be about $11.55 from our study. This is a conservative estimate, as we did not include potential cost savings when physicians asked their clinical colleagues for the answer or the cost savings, from ordering fewer tests or diagnostic images.

The average cost per question is $38.2 in our study and would be $5.7 if the librarians worked at a 90% utilization rate. The economically break even of the JIT librarian consultation service will require just over twice the utilization rate we used in the JIT service. Thus, the possibility to reap the economic benefit from JIT service is tightly linked to the efficiency of the clinical librarians' team.

## Discussion

This study demonstrates the potential of a JIT service to have positive net economic benefit if the involved librarian team are efficiently organized and managed. It is difficult to decide the best way to determine costs for a librarian consultation service. We chose to do a CCA to target health decision-makers as we felt this method would allow them determine how to value a librarian consultation service and decide if this service is good value for its cost.

The use of CCA in the field of library and information science is uncommon. In a review of other clinical question and answer series, only two of the six papers discussed costs [7]. One did not indicate what costs were included in the service so there was no way to compare [23]. The other indicated that a formal health economic assessment is needed to compare the costs of the service with the costs and benefits of altered patient care resulting from the answers and indicated that it is possible that a team approach - for example, librarian/information specialist plus clinical epidemiologist and administrative support - would produce more rapid and cost-effective answers to a larger group of practitioners [12]. This suggestion of a team approach with administrative support is supported by this project. Weightman and Williamson found in a systematic review that two clinical librarian studies showed evidence of cost effectiveness [24].

Our results from the CCA showed that the cost per question for JIT was quite low and could be reduced further. Implementing a service like this nationally could happen quickly as the time required to train professional librarians to do this service is short. It is estimated that for the same workload, an ongoing project could require fewer trained librarians, which would reduce labor costs further. Having librarian work remotely is another option that could be used. Benefit of librarians working remotely include decreased (or eliminated) office costs as well as allowing the service to cover different time zones without incurring overtime costs.

The use of librarians versus primary health care professionals to locate information to answer clinical questions was less costly. If you consider the cost for 15 minutes of librarian time, the average salary cost in the project was approximately $7.15 (based on 15 minutes), while the average salary cost for 15 minutes of a FGH or FHN physician ranges from $20.75 to $27.69. It is not surprising that librarians are less costly than physicians. Additionally, a librarian is an appropriately trained professional to conduct literature searches.

The cost of the hand-held is another area that could be investigated further. In a participant satisfaction questionnaire about using the librarian consultation service, when asked about preferred methods to send requests, 43.1% indicated the web and 38.9% indicated hand-held. The direct costs for the hand-held devices and monthly fees were significant. Further, the financial management related to the hand-held devices was a significant task in the project. Using the Internet exclusively or a combination of hand-held and Internet could reduce costs and workload significantly.

Physicians have a lot of unanswered questions and that they do not pursue the majority them [4], [6]. This is also clearly true is our study where physicians only asked about 2 questions a month. Further information and research is needed to understand why or whether or not it matters. It may be because it is possible for the FP to manage the patient without knowing the answer to the question. It may not be in the most patient centered approach, but perhaps it is enough to address the reason for the visit. It is recommended that if another iteration of the service were to proceed, it's design should focus on the ease of use of the service to increase the number of questions asked by physicians.

### Limitations

We did not differentiate the types of clinical questions (diagnosis, etiology, preventive, prognosis, therapy) and the difficulty level of the question in our analysis. Although we took conservative approach in using the benefit schedule of health insurance in Ontario to estimate the average hourly wage rate for the FP and the average cost for a visit to a specialist, the arbitrariness in choosing the service list for estimating the average cost still exists. We have no data to indicate that physicians will choose to see an extra patient when they save approximately 20 minutes of time in the middle of their day. Certainly if the time were saved near the end of their day, there would be no opportunity to bring in a “replacement” patient. Physicians working in group practice may find it easier to see additional patients.

A literature review by Coumou and Meijman looked at twenty-one original articles and three literature reviews to try to answer how primary care physicians seek information, their strategies, the time required, their evaluation strategies and whether or not a librarian could be used for some of these tasks [25]. They found large variation in the number of questions posed and in the percentage of questions that actually led to information seeking.
